# The role and mechanism of inflammatory response to growing rod implantation in early onset scoliosis

**DOI:** 10.3389/fcell.2023.1282573

**Published:** 2023-10-26

**Authors:** Haoran Zhang, Bingtai Han, Zhiyi Li, Yiwei Zhao, You Du, Yang Yang, Shengru Wang, Jianguo Zhang

**Affiliations:** Department of Orthopedic Surgery, Peking Union Medical College Hospital, Peking Union Medical College and Chinese Academy of Medical Sciences, Beijing, China

**Keywords:** growing rod, early onset scoliosis, metalosis, inflammatory response, NLRP3 inflammasome

## Abstract

Growing rod implantation, a surgery treatment for EOS (early onset scoliosis), may cause a kind of chronic inflammation called metalosis and all other implant-related complications because of the metal debris released by the implants as a result of fraction and corrosion. There is no complete explanation of immunologic mechanisms of metalosis up to now. This review demonstrates the researches on metalosis from the clinical issues down to basic immunologic mechanisms. Adverse reactions of metal implants are mainly the formation of NLRP3 (nod-like receptor protein 3) inflammasome, primed by TLR4 (toll-like receptor protein 4), activated by phagocytosis and often accompanied by type Ⅳ hypersensitive reaction. Recent studies found that TNF-α (tumor necrosis factor α) also participates in priming, and activation of inflammasome requires disturbance of lysosome and release of cathepsin B. Ca-074Me and MCC950 are therapeutic interventions worth exploring in aseptic loosening of orthopedic implants.

## 1 Introduction

EOS refers to scoliosis in young children, characterized by severe deformities, rapid progression, and often affects thoracic development, leading to respiratory dysfunction. In clinical practice, different treatments are recommended based on the severity of scoliosis: for patients with Cobb angles less than 30°, rehabilitation training or wearing braces can be used (Dede and Sturm, 2016); while for patients with scoliosis exceeding 30°, spinal implants can be considered, and the use of growth rods can effectively control the progression of scoliosis while preserving the growing capacity of the spine, thereby ensuring normal respiratory function development. The indications for growing rod implantation also include: the patient’s age should be between 5 and 10 years old, and being too young can lead to spontaneous fusion, but not being old enough for fusion surgery. The length of the scoliosis segments should not be too short, otherwise fusion surgery will show better prognosis. After implantation, it is necessary to perform distraction surgery every 6–12 months as the spine grows (magnetic control growing rod about one a month) until the growth and development process is completed, that is, the growing rod graduation. Most patients require spinal fusion surgery at this time to prevent the progression of scoliosis.

The patient’s spinal growth and daily activities may cause micro-friction between the metal implants and the human bone, as well as between the implants themselves, releasing metal particles and ions, resulting in a series of allergic reactions, clinically known as metalosis (Zhang et al., 2020). Such reactions can lead to osteolysis, causing aseptic loosening of pedicle screws, and internal fixation failure in severe cases, one of the main complications. However, screw loosening will aggravate the generation of micro-friction, release more debris and aggravate the process of metelosis, which acts in a positive feedback mechanism.

## 2 Molecular mechanisms of EOS

EOS can be categorized into various types based on different etiologies, including idiopathic, congenital, neuromuscular, and more. Genetic factors play a role in the development of EOS. Among these, research on the genetic etiology of congenital scoliosis (CS) is quite extensive. Studies on spondylocostal dysostosis (SCDO), a condition characterized by vertebral and rib malformations, have provided deeper insights into the genetic factors contributing to spinal segmentation abnormalities. Mutations in genes such as DLL3 (delta-like 3), MESP2 (mesoderm posterior bHLH transcription factor 2), LFNG (O-fucosylpeptide 3-beta-N-acetylglucosaminyl-transferase), HES7 (hes family bHLH transcription factor 7), and TBX6 (T-box 6) lead to the development of different types of SCDO (SCDO I, II, III, IV, V). These five genes are all members of the Notch signaling pathway and play crucial roles in regulating somite formation and development (Sparrow et al., 2012). The Notch signaling pathway is essential for skeletal development through intercellular communication between neighboring cells. Mutations in genes within this pathway, whether causing loss of function or gain of function, can result in severe skeletal disorders (Zanotti and Canalis, 2016).

Apart from these pivotal genes in the Notch pathway, there are other genes considered as important candidate pathogenic genes associated with spinal defects and scoliosis. For instance, mutations in the PAX1 (paired box 1) gene affect vertebral segmentation, and Giampietro et al. reported mutations at different sites of the PAX1 gene in 5 out of 48 CS patients, suggesting its candidacy as a causative gene for CS(Giampietro et al., 2005). The SHOX (short stature homeobox) gene, known for its role in growth and development, has been associated with various skeletal abnormalities, including scoliosis, due to its influence on vertebral development (Day et al., 2009). Hayes et al. conducted research on the ptk7 (protein tyrosine kinase 7) gene in zebrafish and found that functional loss of ptk7 could lead to EOS(Hayes et al., 2014).

## 3 Inflammatory complications of growing rod implantation

Although growing rods have satisfactory effect for EOS patients in controlling scoliosis and preserving growing potential, complications are frequently reported. In addition to trauma, anesthesia, and unexpected surgical injuries during implantation and distraction surgery, there are also complications caused by implant failure (broken rods, loosening of pedicle screws, etc.) and corrosion (inflammation, osteolysis, neurological function damage, etc.). The statistical results of the incidence rate of complications around the world are inconsistent. A retrospective study published on Spine Journal from Virginia Medical Center in the United States included 19360 EOS patients from 2004 to 2007, and the total incidence rate of complications is 10.2%. The highest five complications are deep infection (1.7%), internal fixation failure (1.5%), nerve injury (1.0%), shallow wound infection (1.0%) and lung function injury (1.0%) (Reames et al., 2011). Deep infection is categorized to septic inflammation almost inevitably needs repair, which is not included in this review. As surgical technique develops, especially aseptic technique, the significance of aseptic loosening in growing rod failure is often underestimated. These situations not only exist in patients with growing rod implantation, but also in other spinal internal fixations and even hip joint prostheses with similar adverse reactions.

The immune response mediated by growing rods is characterized as chronic inflammation in the tissue biopsy around the growing rods, which is manifested by macrophage accumulation, skeletal muscle atrophy, accumulation of small black particles in the dense fibrotic area, mild chronic inflammatory cell infiltration in most areas near the black particles, and macrophages containing melanin deposition at high magnification (Zhang et al., 2020). After internal fixation surgery, an increase in metal ion concentrations can be detected in patients, such as titanium, chromium, nickel and other metal ions (Siddiqi et al., 2021).

## 4 Mechanism of inflammatory response to growing rod implantation

The immunological mechanisms of adverse reactions mediated by metal implants are still not illustrated completely. Our interpretation about metal-induced inflammation has gone through a tortuous process. Initially, adverse reactions to metal implants were believed to be caused by microorganisms infected during surgery. With the progression of research on pattern recognition receptors (PRRs), it has been proved that bacteria contain pathogenic-associated molecular patterns (PAMPs) such as lipopolysaccharides (LPS), which can act as modulators of metal particles and prompt macrophages to differentiate into pro-inflammatory subtypes (Mosser and Edwards, 2008). This discovery strictly divides the inflammatory responses to metal implants into septic and aseptic ones. After the development of more advanced sample processing and detection technologies, some cases that supposed aseptic proved to be bacterial, such as bacteria being able to adhere to the surface of implants in the form of biofilms. Therefore implant-related bacterial infections are more common than estimated (Konttinen and Pajarinen, 2013). However, as aseptic technique in surgery become more secure today, the incidence rate of bacterial infections is gradually decreasing, and aseptic inflammation mainly caused by metalosis have once again become the focus of our attention.

The mechanism of metalosis includes the following aspects: 1) metal ions entering the blood reach important organs and cause damage; 2) local inflammatory response; 3) osteolysis resulting in aseptic loosening of metal implants, leading to internal fixation failure. Moreover, loosening of screws increases micro-friction and releases more debris. This review mainly discusses the mechanism of inflammation, that is, the immune response caused by metal implants. Currently, some researchers suggest that this is an inflammatory process featuring NLRP3 inflammasome (Konttinen and Pajarinen, 2013). In recent years, there has been sufficient research on NLRP3 inflammasome, which includes two stages: priming and activation. The former requires the participation of molecules like LPS, TNF- α and IL-1 β, while the latter relies on various pattern molecules that can be recognized by the natural immune system, and the intracellular signals downward include K^+^ efflux, Ca^2+^ flow, lysosomal destruction, and the production of mitochondrial oxygen free radicals. After activation, NLRP3 is assembled into complete inflammasomes and caspase-1 is activated, the later activating IL-1 β and IL-18. Simultaneously the inflammasomes shear gasdermin D and insert it into the cell membrane, forming pores and inducing apoptosis, releasing intracellular pro-inflammatory cytokines (Swanson et al., 2019). In the inflammation caused by metal implants, TLR4 binds to metal ions to mediate the priming process, and the phagocytosis of metal particles mediates the activation stage. CD4^+^ T cells then recruit more inflammatory cells in the infected area to trigger type IV hypersensitivity response (Konttinen and Pajarinen, 2013).

Titanium alloy, the most commonly used material of growing rods as well as other orthopedical instruments, has been investigated in several cytological experiments (Table 1). In 2022, Petterson M. et al. used wild-type THP-1 (wt.) cells and NLRP3 and ASC (apoptosis-associated speck-like protein containing caspase recruitment domain (CARD)) knockdown cells to be treated with both macro- and nanoparticles of Ti and TiO_2_, and found that cells exposed to the Ti-ion solution showed a dose-dependent increase of IL-1β release, but exposure to particulate Ti did not result in increased IL-1β release (Pettersson et al., 2022). Similar result has been reported in 2020 by Xiao Li et al., 2020 in Jurkat T cells, who also indicated that Ti ions can promote the production of ROS, while NLRP3 expression and IL-1β secretion are reduced after treatment of Jurkat cells with NAC (ROS scavenger). Other materials have also been studied. In 2023, F. Brunken showed that CoNiCrMo particles could activate more inflammasome than TiAlV particles, indicating that different inflammatory pathways are activated by different alloys (Brunken et al., 2023).

**TABLE 1:** Present studies regarding Ti alloy in NLRP3 inflammasome activation and its potential alleviative targeting drugs.

Year	Author	Activator/Alleviator	Mechanism	Model	Reference
2020	X. Li	Ti ions	Promote Ros production	Human Jurkat T cell	Li et al. (2020)
2010	C. A. St Pierre	Ti particles	Lysosome and lysosomal cathepsins	Murine and human primary macrophages, THP-1 cells	St Pierre et al. (2010)
2017	F. Jessop	Ti nanoblets	Lysosome membrane permeabilization increase and cathepsin leakage	Caspase-1-deficient mice, mouse bone marrow derived macrophages	Jessop et al. (2017)
2021	K. Zheng	Ti particle	GSK-3β/β-catenin pathway	Male Sprague–Dawley rats, rat mesenchymal stem cells	Zheng et al. (2021)
2022	M. Pettersson	Ti ions	Induce release of bioactive IL-1β	Monocytic leukemia cell line THP-1	Pettersson et al. (2022)
2019	R. C. Coll	MCC950	Block ATP hydrolysis	C57BL/6 and Asc−/− mice and human peripheral blood mononuclear cells	Coll et al. (2019)
2021	Y. Wu	Melatonin	Activate butyrate/GPR109A signaling pathway	C57BL/6 J male mice	Wu et al. (2021)

### 4.1 The role of innate immune cells

Distinct issues linked to growing rods have been recognized, encompassing challenges such as metallosis (Cheung et al., 2016). Metallosis is defined as aseptic fibrosis, local necrosis, or loosening of a device secondary to metal corrosion and release of wear debris. When viewed with the naked eye during revision surgery, metallosis manifests as the formation of a pseudo-capsule around the rods, accompanied by an aggregation of black/grey particles. The wear particles around the rods are predominantly composed of titanium and are roughly 3 µm in diameter. The precise components and concentrations of these particles within human tissues remain uncertain (Teoh et al., 2016).

A previous histological study observed chronic inflammatory responses associated with metallosis for patients with the growing rods under low magnification (Teoh et al., 2016). Histological analysis was conducted on tissue samples obtained from the vicinity of the growing rods in the four patients affected by metallosis. Microscopic examination consistently revealed the presence of black and grey granular particles, hyalinized fibrous tissue, as well as a chronic inflammatory response marked by infiltrations of lymphoid and plasma cells.

Titanium particles are important as they activate fibroblasts and macrophages. Biopsies were performed on paraspinal muscles from ten patients with EOS who underwent growing rods exchange (Zhang et al., 2020). Microscopic examination of the H&E stained specimens indicated that the samples primarily consisted of fibromuscular tissues, with some instances of atrophic skeletal muscle. Notably, an accumulation of fine, black particles was observed in the dense fibrotic areas. Additionally, there was evidence of a mild active and moderate chronic inflammatory cell infiltration in most of the slides near the black particles. Under high magnification, macrophages containing black pigmentations were discernible. It's important to note that no instances of local neoplasia were detected during the examination.

### 4.2 Pattern recognition receptor binds metal ion to mediate immune response

TLR is one of the most typical pattern recognition receptors, which is a dimer with three structural domains: extracellular domain, transmembrane domain, and intracellular domain. The most important pattern molecule recognized by TLR is bacterial LPS. Recent researches prove that the membrane type Toll-like receptor TLR4 can also be activated by metal ions. Genetic defect testing at different TLR4 loci shows that non-conserved his456 and his458 are necessary for metal ion binding (Schmidt et al., 2010), but do not overlap with the binding site of LPS. Activation of TLR4 by metal ions is also less than that of LPS (Tyson-Capper et al., 2013). Previous research has demonstrated the exceptional potency of LPS, which can elicit host responses even at femtomolar concentrations, equivalent to roughly 100 invading Gram-negative bacteria (Lajqi et al., 2019; Lajqi et al., 2020). Mechanistically, the activation of immune cells by LPS is mediated through TLR4. This receptor activation hinges on the binding of a single LPS molecule to the MD-2/TLR4 dimeric receptor. Notably, as few as 25 LPS MD-2.TLR4 complexes per cell can trigger measurable pro-inflammatory responses, underscoring the highly efficient oligomerization of these ternary complexes (Teghanemt et al., 2013). Moreover, the ability of TLR4 to be activated by metal ions is different among species, and no similar binding ability was found in mice (Schmidt et al., 2010).

After TLR4 is activated, the downstream molecular mechanism is consistent with that induced by LPS, connecting the junction protein through the intracellular domain, activating protein kinase, producing different transcription factors, and releasing cytokines mainly composed of pro-inflammatory factors. Its pathways are divided into MyD88 dependent and MyD88 independent. The former is regulated by nuclear transcription factors AP-1 and NF-κB and then initiates the expression of target genes and mediates the production of inflammatory cytokines, while the latter induces phosphorylation and nuclear translocation of interferon regulatory factor 3 (IRF3), thereby regulating the production of type I interferon and inflammatory cytokines. NF-κB initiates target gene to express IL-1β, an effective pro-inflammatory factor, and NLRP3 molecule, which are involved in the priming of NLRP3 inflammasome.

In addition to priming by TLR4, E. Jämsen et al., 2020 demonstrated particle-induced IL-1β depends on the activation of NLRP3 inflammasome *in vitro* experiments by using specific inhibitors targeting the inflammasome signaling pathway. Furthermore, it was demonstrated that tumor necrosis factor (TNF) can replace LPS and participate in the initiation stage of NLRP3 inflammasomes. This discovery provides a more reasonable explanation for the large production of inflammatory bodies in sterile environments. TNF is the transcription product of NF- κB during the initiation stage, enhancing its own initiation process, while the metal ions involved in the initiation mentioned earlier are very low in content and only initiate the initiation process of inflammatory bodies. However, the activation level of TNF is also much lower than that of classical LPS. In addition, this study used *in vitro* experiments, and the dose was 1000 times higher than physiological conditions, which cannot guarantee the same results to be repeated *in vivo* (Jämsen et al., 2020).

Innate immune responses to subsequent injury may also be a source of long-term inflammatory responses after growing rods implantation. Trained immunity, refers to a lasting functional reprogramming triggered by a range of factors. These factors can include endogenous danger signals known as damage-associated molecular patterns, which are released during cellular stress or tissue injury, as well as exogenous conserved molecules from pathogens called pathogen-associated molecular patterns (Braza et al., 2018; Lajqi et al., 2023). Trained cells undergo a sustained activated state marked by heightened production of pro-inflammatory mediators like IL-1β, TNF-α, IL-6, as well as increased levels of reactive oxygen species. This activation is accompanied by enhanced antimicrobial and antitumoral capabilities, primarily orchestrated through epigenetic modifications leading to alterations in metabolism and functional reprogramming (Netea and Joosten, 2018).

Trained immunity signifies a functional state observed in innate immune cells and tissue-resident stem cells, where they adapt their responses to subsequent insults (Lajqi et al., 2021). This adaptation is notably characterized by a sustained activation following a period of rest, primarily orchestrated through epigenetic reprogramming and metabolic rewiring in cells (Ferreira et al., 2022). It reflects the innate immune system’s ability to generate memory-like responses against both past microbial and non-microbial challenges. Current research indicates that trained immunity can persist in innate immune cells for extended periods, ranging from months to even decades (Jentho et al., 2021).

### 4.3 The phagocytic effect of macrophages on metal particles mediates the activation of NLRP3 inflammasomes

**FIGURE 1:**
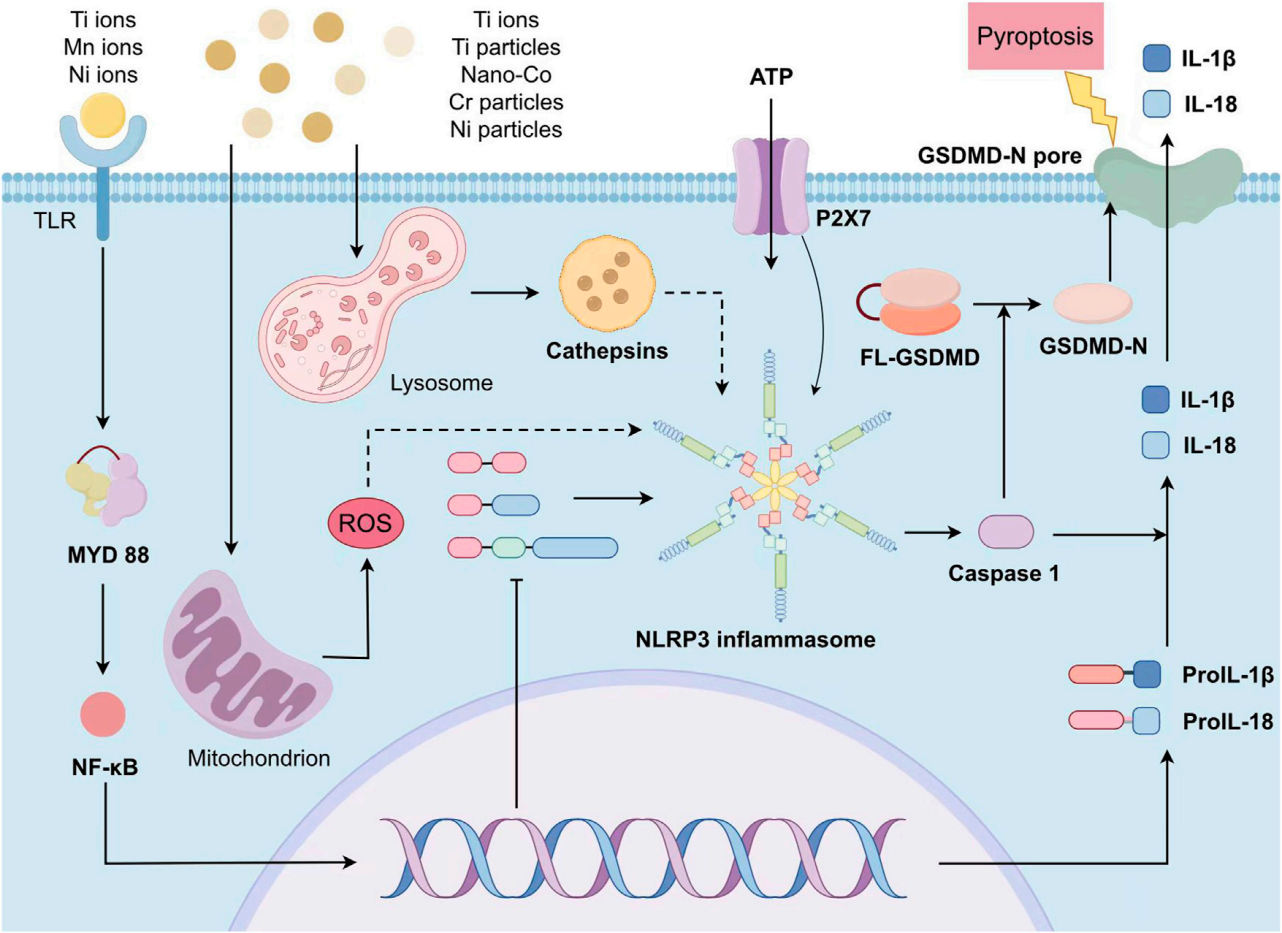
The scheme of metal-induced activation of NLRP3 inflammasome. The priming stage requires the participation of metal ions and cytokines as IL-1 β. The activation stage relies on the metal-activated intracellular signals including ATP intake, K+ efflux, Ca2+ flow, lysosomal destruction, and the production of mitochondrial oxygen free radicals. After activation, NLRP3 is assembled into complete inflammasomes and caspase-1 is activated, producing IL-1 β and IL-18. Simultaneously the inflammasomes shear gasdermin D and insert it into the cell membrane, forming pores and inducing pyroptosis, releasing intracellular pro-inflammatory cytokines.

Before the metal particles interact with cells, they need to undergo modulation effect. Common opsonin includes immunoglobulin, lectin, and complement fragments such as C3b, and iC3b. The metal particles enter macrophages through modulatory phagocytosis, and the rupture of phagosomes releases them into the cytoplasm, activating NLRP3 inflammasomes. The specific mechanism has not yet been elucidated. In addition, excessive metal particles in cells can lead to cell apoptosis and release intracellular substances after rupture. As a damage associated molecular pattern (DAMP), it can also participate in the activation of NLRP3 inflammasomes (Figure 1).

Phagocytosis and lysosomal breakdown and release of cathepsin B can activate NLRP3 inflammasomes. In 2008 V. Hornung found through *in vitro* that NLRP3 inflammasomes can be activated by silicon crystal particles, leading to phagocytosis of crystals and subsequent lysosomal damage and rupture. Sterile lysosomal damage can also lead to activation of inflammasomes, while phagocytic acidification or inhibition of cathepsin B activity can impair the activation of NLRP3 inflammasomes, which indicates that NALP3 inflammasomes perceive lysosomal damage as an endogenous danger signal (Hornung et al., 2008). However, this study did not distinguish between phagocytosis and cell death, and silicon crystal particles may not necessarily stimulate cells to produce the same response as metal particles. One experiment conducted by Jessop et al., 2017 indicated that the lysosomal vATPases inhibitor Bafilomycin A1 blocked lysosome membrane permeabilization *in vitro* and *ex vivo* in primary murine macrophages following exposure to titanium nanobelts, which proved the participance of Ti particles in this same pathway as silicon. In 2021 B.P. Fort et al., 2021 used wild-type and gene manipulated mouse macrophages and drug inhibitors and found that NLRP3 and gasdermin D were necessary for the release of IL-1β induced by metal particles, rather than particle induced cell death, which proved that these processes occur independently. Phagocytosis and release of lysosomal cathepsin are crucial to both IL-1β production and cell death, none of which is dispensable.

The assembly process of NLRP3 inflammasome has been extensively studied in recent researches. Multiple molecular and cellular signaling events mentioned before are involved. Moreover, in 2021, Kai Z. et al. revealed that upregulated SIRT3 (Sirtuin3) dramatically reduced Ti particle-induced osteogenic inhibition through suppression of the NLRP3 inflammasome in the process of Ti particle-induced osteolysis relying on the GSK-3β/β-catenin signaling pathway (Zheng et al., 2021). However, none of these is a common event induced by all the NLRP3 inflammasome stimuli.

### 4.4 Type IV hypersensitivity response triggers stronger and more persistent inflammation

Metal ions released by metal implants themselves and corroded by metal particles in acidic environments of phagocytic bodies can serve as allergens for type IV hypersensitivity response. At present, metal ions have not been used as a model molecule. After metal ions enter the human body, there are three sensitization methods: binding to native peptides as haptens, directly binding to MHC class II molecules and altering their conformation to be recognized as allogeneic cells, or binding as a superantigen to MHC class II molecules and T cell receptors (TCRs) in a non-peptide dependent manner, producing strong sensitization effects (Gamerdinger et al., 2003). The ability of metals to produce these new antigens demonstrates the similarity between metal-induced hypersensitivity reactions and autoimmunity (McKee and Fontenot, 2016).

The type IV hypersensitivity reactions caused by metals in the human body include three phases: recognition phase, activation phase, and effect phase. CD4^+^ T cells recognize antigens that are processed and presented by antigen-presenting cells (dendritic cells, also M cells, monocytes, and even vascular endothelial cells), known as recognition phases. Th cells that have been exposed to antigens migrate to the infected site and get activated. Due to the scarcity of these specific cells and the relatively mild inflammation of the lesion, it takes several hours for T cells to arrive. These cells release cytokines, activate local endothelial cells, recruit inflammatory cells dominated by macrophages for infiltration, and lead to accumulation of fluid, plasma proteins, and more white blood cells, forming a visible lesion known as the effector phase. Murine experiments have shown that this hypersensitivity response runs in a sex and age-related manner. Samelko L. et al. proved in 2021 that young female mice are more predisposed to metal-augmented inflammatory responses to wear debris, which is highly influenced by active NLRP3 inflammasome/caspase-1 signaling pathway (Samelko et al., 2021).

## 5 Discussion

Metalosis is one of the most important complications of growing rod implantation surgery, and may secondary to various complications related to internal fixation failure. Therefore, studying its immunological mechanism and taking countermeasures may be a potential strategy to reduce the incidence rate of complications. Any interference against its pathway would serve as a novel type of immunosuppression drug. In 2019, R. C. Coll proved that MCC950 directly interacts with the Walker B motif within the NLRP3 transcription region to block ATP hydrolysis and inhibit NLRP3 activation (Coll et al., 2019). In 2022, Lin S. et al. demonstrated that lncRNA Neat1 is a key regulator in regulating wear particles-induced osteolysis by activating NF-κB pathway, NLRP3 inflammation and M1 polarization via Bruton’s tyrosine kinase (Lin et al., 2022), which may act as a potential therapeutic target for metalosis. Wu et al., 2021 revealed that melatonin also alleviates this inflammatory response via activation of butyrate/GPR109A signaling pathway, which depends on modulating gut microbiota and regulating butyrate production. However, there is still little research on its pathogenic mechanism, and there is no complete explanation or specific drug. Although surface modification effectively reduces the occurrence of infection, the risk still exists. At present, the core research of spinal surgeons is on analyzing genetic mechanisms, establishing more detailed spinal classification, optimizing surgical plans, improving surgical instruments and so on. These projects are more effective in reducing complications at this stage and are currently supposed to be urgent problems to be solved. However, the clinical significance of metalosis itself is not as important as many of the work above. In the future, when surgical techniques reach a higher level and people have higher expectations for the prognosis of growing rod surgery, more attention may be paid towards adverse reactions of metal implants, further reducing the occurrence of complications.

In addition, the impact of metal debris retention in patients with growing rods after graduation on their physical condition in adulthood is a blank, and long-term follow-up is needed for these patients. This review mostly draws on other metal implant surgeries such as hip joint replacement, and the majority of patients with hip joint replacement are middle-aged and elderly people. EOS patients are children aged 5–10 years old, and their physiological conditions may not be completely the same as those of adults, such as metal content in the body, tolerance to metal generated antigens, degree of osteolysis, etc. These data also require future statistical analysis of a large sample to supplement.
